# Editorial: Challenges and advances in primary antibody deficiencies diagnosis, treatment and follow-up

**DOI:** 10.3389/fped.2025.1702168

**Published:** 2025-09-25

**Authors:** Adriano La Vecchia, Lucia Augusta Baselli, Carlo Agostoni, Rosa Maria Dellepiane

**Affiliations:** ^1^ Pediatric Area, Fondazione IRCCS Ca’ Granda Ospedale Maggiore Policlinico, Milan, Italy; ^2^ Department of Medicine and Surgery, University of Milan-Bicocca, Monza, Italy; ^3^ Department of Clinical Sciences and Community Health (DISCCO), University of Milan, Milan, Italy

**Keywords:** antibody deficiencies, MIS-C (multisystem inflammatory syndrome in children), IVIG = intravenous immunoglobulin, Top2 (Topoisomerase 2), genetic advance, CVID- common variable immunodeficiency disorders, glucocoricosteroids, XLA agammaglobulinemia

**Editorial on the Research Topic**
Challenges and advances in primary antibody deficiencies diagnosis, treatment and follow-up

## Introduction

Immune dysregulation, whether due to deficiency or hyperactivation, results from a complex interplay of genetic, environmental, and infectious factors. Recent progress in genomic diagnostics, newborn screening, and mechanistic studies has dramatically expanded the recognized spectrum of inborn errors of immunity, which now includes more than 550 distinct genetic defects (1–3). Innovations in biologic therapies and advanced monitoring tools such as multiple breath washout (4) and magnetic resonance imaging (5) are transforming how pediatric immunodeficiencies and inflammatory disorders are diagnosed, managed, and prognosed.

At the same time, globalization and emerging infections have introduced new challenges. The Severe acute respiratory syndrome coronavirus 2 (SARS-CoV-2) pandemic has highlighted both acute and long-term implications for immune regulation (6). Beyond the emergence of Multisystem Inflammatory Syndrome in Children (MIS-C), children with inborn errors of immunity are at heightened risk for severe coronavirus disease 2019 (COVID-19) and may exhibit suboptimal vaccine responses (1). Reduced exposure to common pathogens during lockdowns raises questions about long-term immune maturation, allergy, and autoimmunity, consistent with the Old Friends hypothesis (7). Integrating genomic insights with post-COVID immunology may allow earlier identification of children at risk for severe or atypical immune responses.

This Research Topic brings together contributions that illustrate how genomic medicine and post-COVID immunology are reshaping pediatric immunology.

## Genetic advances

Common variable immune deficiency (CVID) and X-linked agammaglobulinemia (XLA) remain the most frequent predominantly antibody deficiency disorders. Traditional genetic methods, such as Sanger sequencing of the tumor necrosis factor receptor superfamily, member 13b/transmembrane activator and CAML interactor (TACI/TNFRSF13B) in CVID and Bruton's tyrosine kinase (BTK) in XLA, continue to confirm diagnoses and uncover pathogenic variants.

Chakrobortty et al. conducted a cross-sectional study in Bangladesh of 35 children with suspected predominantly antibody deficiencies. They used serum immunoglobulin measurement, immunophenotyping, Polymerase Chain Reaction (PCR), and Sanger sequencing of BTK and TACI. Among 15 confirmed cases, 7 had CVID, 7 had XLA, and 1 had another form of agammaglobulinemia. BTK sequencing identified seven pathogenic or likely pathogenic variants, three of which were novel, while no pathogenic TACI variants were detected. This study demonstrates how implementing genetic testing in countries where it is not routinely available can improve diagnosis and patient care.

Zhu et al. used whole-exome sequencing to uncover a novel topoisomerase II beta (TOP2B) variant in an infant with epileptic spasms, B-cell immunodeficiency, and dysmorphic features. This case shows how sequencing technologies can reveal previously unrecognized genotype-phenotype associations and guide targeted therapy.

Boyarchuk et al. described two neonates in Ukraine with B-cell lymphopenia identified through newborn screening. Both carried Immunoglobulin lambda-like polypeptide 1 (IGLL1) variants, c.425C>T and c.258del. Although their clinical manifestations were mild and immunoglobulin levels were near normal, early identification enabled close monitoring and timely therapy, preventing severe complications. These cases highlight how newborn screening can reveal underdiagnosed causes of B-cell lymphopenia. Unfortunately, healthcare disruptions in conflict zones limit access to genetic testing and early interventions (8).

Together, these studies illustrate how targeted gene analysis, whole-exome sequencing, and newborn screening are expanding the genetic and clinical spectrum of predominantly antibody deficiencies. They also show how early diagnosis can improve management worldwide. Looking ahead, whole genome sequencing, single-cell immune profiling, and gene therapy are expected to further transform care.

## New treatment approaches for immune dysregulation

Advances in understanding the genetic and immunologic basis of immune dysregulation have enabled more targeted therapies. Immunoglobulin replacement remains the cornerstone for antibody deficiencies and related rare disorders, as demonstrated by Zhu et al., where early intervention prevented severe infections in a child with a novel TOP2B defect.

Biologic therapies are also showing promise for complex complications. Xie et al. reported a juvenile patient with chronic graft vs. host disease and refractory arthritis who improved markedly after intra-articular tocilizumab injections. This resulted in better joint function and lower interleukin 6 levels, showing how biologics may be repurposed in challenging situations.

In the setting of MIS-C, Lin et al. performed a meta-analysis of 4,269 patients. They found that glucocorticoid monotherapy significantly lowered treatment failure and persistent fever, while combination therapy with intravenous immunoglobulin reduced the need for further immunomodulation. These results highlight the importance of evidence-based approaches guided by genetic and immunologic profiling.

Additional modalities such as Janus kinase (JAK) and phosphatidylinositol 3-kinase (PI3K) inhibitors, hematopoietic stem cell transplantation, and gene therapy are expanding treatment options for genetically defined inborn errors of immunity (1). Careful application of these strategies, tailored to individual context, will continue to improve outcomes.

## Global health and equity considerations

Scientific advances promise transformative benefits, but healthcare disparities limit their global impact. The experiences from Bangladesh and Ukraine emphasize the importance of equitable access to genomic testing, newborn screening, and biologic therapies (1, 9). Strengthening healthcare systems and investing in scalable genomic and therapeutic technologies are essential to translate scientific progress into better outcomes for children worldwide.

## Conclusion

Genetic diagnostics, newborn screening, and precision therapies are redefining the landscape of pediatric immunodeficiencies and inflammatory disorders. These innovations enable earlier diagnoses, guide targeted treatments, and support long-term management, improving outcomes globally. At the same time, the COVID-19 pandemic, global inequities, and geopolitical instability highlight the need for equitable healthcare strategies. Continued investment in genomic technologies, immunomodulatory therapies, and infrastructure, combined with international collaboration, will be essential to ensure that every child with an immune disorder benefits from these advances.
